# Efficacy of Breast Milk Olfactory and Gustatory Interventions on Neonates’ Biobehavioral Responses to Pain during Heel Prick Procedures

**DOI:** 10.3390/ijerph19031240

**Published:** 2022-01-22

**Authors:** Chiao-Hsuan Lin, Jen-Jiuan Liaw, Yu-Ting Chen, Ti Yin, Luke Yang, Hsiang-Yun Lan

**Affiliations:** 1 Department of Nursing, Tri-Service General Hospital, Taipei 11490, Taiwan; 498010365@mail.ndmctsgh.edu.tw (C.-H.L.); eden@mail.ndmctsgh.edu.tw (T.Y.); 2 School of Nursing, National Defense Medical Center, Taipei 11490, Taiwan; jiuan@mail.ndmctsgh.edu.tw; 3 Graduate Institute of Medical Sciences, National Defense Medical Center, Taipei 11490, Taiwan; 804010023@mail.ndmctsgh.edu.tw; 4 Department of Social Work, Hsuan Chuang University, Taipei 30092, Taiwan; LHY@hcu.edu.tw

**Keywords:** breast milk odor and taste, olfactory and gustatory interventions, neonates, pain, biobehavioral response, syringe feeding, heel prick

## Abstract

This study aimed to evaluate the efficacy of breast milk odor either alone or in combination with breast milk taste (via syringe-feeding) to alleviate neonates’ biobehavioral responses to pain during heel-prick procedures. This prospective randomized controlled trial recruited 114 neonates by convenience sampling from a newborn unit of a medical center in Taiwan. Neonates were randomly assigned to three groups: control (gentle touch + human voice), control + breast milk odor, and control + breast milk odor + breast milk taste. Heart rate, oxygen saturation, and voice recordings of crying were measured across heel-prick procedures: baseline, no stimuli (stage 0); during heel prick (Stages 1–4); and recovery (Stages 5–10). Generalized estimating equations and Kaplan–Meier survival analysis compared differences in changes between groups for heart rate, oxygen saturation, and time to crying cessation. Changes in mean heart rate and oxygen saturation in neonates receiving breast milk odor or breast milk odor + breast milk taste were significantly less than those at the corresponding stage for the control group. Among neonates receiving breast milk odor or breast milk odor + breast milk taste, hazard rate ratios for crying cessation were 3.016 and 6.466, respectively. Mother’s breast milk olfactory and gustatory interventions could stabilize the biobehavioral responses to pain during heel prick procedures in neonates.

## 1. Introduction

Healthy neonates can undergo more than five painful procedures in a hospital’s newborn unit [1]. However, pain in newborn infants is still underassessed and undertreated [2,3]. Although infants are unable to verbally express pain, they can respond to pain through the changes of the physiological parameters and behaviors [4]. Preventing the changes of biobehavioral responses to painful procedures in neonates is a moral obligation for clinicians [1,5]. Although an understanding of infant pain continues to improve pain management, management of the changes of biobehavioral responses to short-term painful procedures has not been a focus [6]. The repeated exposure to pain can have negative short- and long-term effects [7], including adverse physiological, psychological and emotional consequences, [8,9], changes in pain threshold, and prolonged hyperalgesia, which have been associated with impaired brain development in later life [4]. A shift in the focus of neonatal research and clinical practice to advance management of short-term procedural pain could improve care quality [10] and promote long-term infant health and wellbeing [11].

### 1.1. Background

Neonatal pain can be observed through biological responses (heart rate and oxygen saturation) and behavioral responses (crying, changes in facial expression, and changes in body movements) [12], which can be employed as indicators of pain when assessing the effect of analgesic interventions [8]. The use of non-pharmacologic interventions in pain management in infants has become more common due to their availability, accessibility, cheapness, and effectiveness [13,14,15,16,17].

Studies have demonstrated the effectiveness of nonpharmalogical interventions in reducing procedural pain. Single sensory interventions include oral sucrose [14], nonnutritive sucking (NNS) [15], facilitated tucking [16] and swaddling [17]. Multisensory interventions include breast milk (BM) plus swaddling [17], BM plus NNS [18], and sucrose combined with massage, music, NNS, and gentle touch (GT) [19]. For newborn infants, however, the Baby-Friendly Hospital Initiative (BFHI) advocates only feeding BM and forgoing other food or drink except when medically indicated (step 6) and advises against the provision of artificial teats or pacifiers for breastfeeding (step 9) [20]. Therefore, oral sucrose or pacifier sucking are not appropriate interventions for pain relief in breastfed newborns. The BFHI policy guided us to use BM different sensory stimuli to prevent the changes of biobehavioral response to painful procedures.

The olfactory and gustatory senses are fully developed at birth, which allows neonates to comfort themselves when stressed through their sensory capacities [21] and to respond positively to olfactory stimulation associated with their mother’s BM [22]. BM is a natural food that is beneficial and nutritious to an infant [23]. BM contains various macronutrients (carbohydrates, proteins, lipids, and vitamins) as well as numerous bioactive compounds such as growth factors, hormones, immunoglobulin A, and antimicrobial compounds [24]. Infants who are fed with BM have a reduced risk of infections and long-term benefits for the infant development and health [25]. Furthermore, BM is composed of lactose, which has a sweet taste, and tryptophan, which may facilitate secretion of endogenous opioids for buffering biobehavioral pain responses [26]. BM odors reduced changes of heart rate and oxygen saturation of preterm infants during venipuncture compared with infants receiving a vanilla odor [27]. Breastfeeding infants held by mother had reduced pain scores, lower crying duration and heart rate, and higher oxygen saturation during vaccine injections compared with infants receiving a control condition [28]. However, many mothers are exhausted postpartum and may be unable to breast feed their infant when a heel-prick procedure is required. Providing the taste of BM could be facilitated by syringe feeding when mothers are not able to breast feed during painful procedures.

Studies have suggested that multisensory stimulation (BM taste, touch, and sound) may generate analgesic effects during short painful procedures [29]. The mechanism is a reduction in the pain response through gentle stimuli (massage or BM taste) by a descending cascade involving activation of inhibitory pathways, secretion of endorphins, and modulation of the response to noxious stimuli in the spinal cord, called “gate control,” through intermediate interneurons [30]. Research suggests that playing a recording of the mother’s voice can significantly lower pain scores, stabilize heart rates, and shorten crying time in preterm infants during heel-prick procedures [31]. In addition, Fitri et al. [32] reported that multisensory stimulation (sucrose + touch + sound or BM + touch + sound) was more effective in relieving procedural pain than unimodal stimulation (oral 24% sucrose only).

The findings of the above studies suggest that multisensory stimulation can buffer pain and the changes of biobehavioral responses to painful procedures in infants. However, one study demonstrated that BM odor and mother’s BM were not effective in reducing pain scores or crying duration compared with a control group [33]. Another study found no significant reductions in observed stress behaviors (facial expression, body movement, and crying duration) in preterm infants receiving their mother’s BM odor + BM taste during venipuncture [18]. A systematic review revealed that most nonpharmacological interventions had only a moderate analgesic effect on procedural pain in infants and did not completely alleviate pain [34]. The inconsistency in the results of the above studies on the effects of BM odor and oral BM on procedural pain motivated the authors to design a multisensory nonpharmalogical intervention that combined BM odor (BMO) and BM taste provided by syringe feeding (BMTSF).

### 1.2. Aims

The study aim was to compare the effects of three multisensory interventions on bio-behavioral responses to heel-prick procedures for newborn screening. Neonates were randomly assigned to one of three interventions: Group 1, GT and human voice = control condition (CC); Group 2, CC + BMO; and Group 3, CC + BMO + BMTSF. We examined differences in the heart rate variation, oxygen saturation (SpO_2_) variation and crying duration among neonates in the three groups during the heel-prick procedures. We hypothesized that, compared with the infants in Group 1 who received GT and human voice, neonates in Groups 2 and 3: (1) would have lower variations in heart rate during and after heel pricks; (2) would have increased SpO_2_ during and after heel pricks; and (3) would have shorter durations of crying during and after heel pricks.

## 2. Materials and Methods

### 2.1. Design

The study was a double-blind, randomized controlled trial with a repeated-measures design. The study had three groups: Group 1, control condition (CC), GT and human voice; Group 2, CC + BMO; and Group 3, CC + BMO + BMTSF. The study followed the CONSORT guidelines for the reporting of the trial [35]. The biobehavioral outcomes included the heart rate variation, oxygen saturation (SpO_2_) variation, and crying duration among neonates during heel-prick procedures.

### 2.2. Sample and Setting

Convenience sampling was used to recruit healthy neonates who were compatible with the inclusion criteria from a newborn nursery at a medical center in Taiwan. Inclusion criteria were as follows: (1) gestational age (GA) ≥ 37 weeks, (2) birth body weight ≥ 2500 g, (3) healthy neonates without any congenital abnormality, and (4) at least one parent agreed to participate.

**Figure 1:**
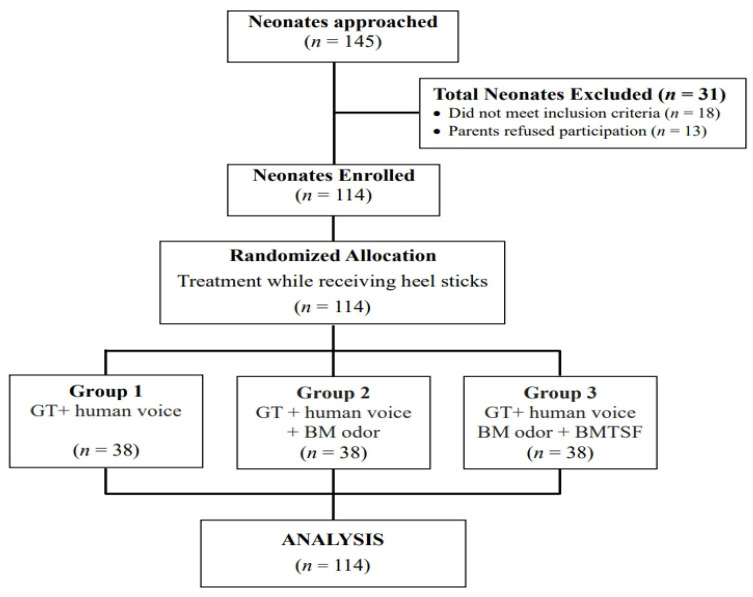
Flowchart of participant recruitment for the three treatment interventions. GT, gentle touch + human voice = control condition; BM, breast milk; BMTSF, breast milk taste by syringe feeding.

A total of 145 infants were screened from September 2017 to September 2018. Among these infants, 18 did not meet the study criteria, and 13 were excluded because the parents did not want to be observed and thus refused to participate (Figure 1). The remaining 114 neonates were randomly assigned to one of three treatment groups using the Sealed Envelope online tool with blocked randomization [36].

The participation rate was 89.76%. Participating neonates did not vary significantly from those whose parents refused participation in terms of GA, birth weight, Apgar score, sex, and delivery type. We used G*Power 3.1.9.2 software (Heinrich Heine University Dusseldorf, North Rhine-Westphalia, Germany) with the “Repeated measures: within–between interactions, MANOVA approach” to assess the sample size required for this study [37]. The study determined effect size in accordance with the results of previous studies [17,38]. By setting effect size = 0.35, α = 0.05, sample size = 114, number of groups = 3, and number of measurements = 11, the estimated study power (two-tailed) was > 0.89.

### 2.3. Measures

Infant demographic and clinical characteristics were obtained by reviewing medical and nursing charts, including GA, birth weight, Apgar score, sex, type of delivery, and number of painful experiences. Painful experiences were defined as any invasive procedures that might cause pain in neonates such as intramuscular injections or blood sugar tests. In the study setting, the infants usually received injections of vitamin K, vaccinations (Hepatitis B), or test of blood sugar within the 24 h after birth. We calculated the total times of painful experiences by summing the procedures in each neonate’s chart. The variables measured were the changes in heart rate and SpO_2_ and time to crying cessation in neonates during and after heel-prick procedures.

#### 2.3.1. Biological Responses to Pain

In this study, biological responses to pain included changes in two parameters of pain: neonatal heart rate and SpO_2_ that were obtained at 1-min intervals using a Masimo Rad 5 Pulse Oximetry monitor. Values were downloaded to a computer by the second author. Certified technicians calibrated the electrocardiographic monitor monthly and checked the computer’s function prior to data collection. The researcher attached an oxygen saturation probe to each neonate’s big toe. Mean baseline heart rates and SpO_2_ levels were compared between the three groups.

#### 2.3.2. Behavioral Response to Pain

The time required for neonates to stop crying (crying cessation) was used a behavioral measure of neonatal procedural pain from baseline to 6 min after the heel prick procedure ended. The definition of crying cessation in our study was the seconds taken to reach the final stop of crying. The neonate’s crying voice was tape recorded using a voice recorder (Sony ICD-UX560F, Tokyo, Japan), which was placed near the neonate’s head. The fourth author blinded to group assignment of the neonates measured the time to crying cessation by listening to the voice recordings for each neonate. A stopwatch was used to mark when the neonate began to cry and was stopped when crying ceases at the final. The time was indicated on a coded form as the time to crying cessation. The corresponding author also listened to the coded recordings and measured the time to crying cessation in the same manner as the fourth author. The interrater reliability for measuring time to crying cessation was 96% for a random sample of 40 neonates.

### 2.4. Data Collection

All data for the neonates were collected after obtaining parental consent. Prior to data collection, the corresponding author trained the researchers involved in this study in how to provide the treatment conditions. The first author was trained as the intervener and received instruction in how to be consistent when providing all sensory interventions as well as how to review and record each neonate’s background data from medical and nursing charts. The fourth author was trained to measure time to crying cessation by listening to the voice recording over the heel-prick procedures. The second author was trained to operate the Masimo Rad 5 Pulse Oximetry monitor to collect biological parameters of pain (heart rate and SpO_2_) and was instructed in how to download and recorded the biological data.

#### 2.4.1. Heel Pricks

**Figure 2:**
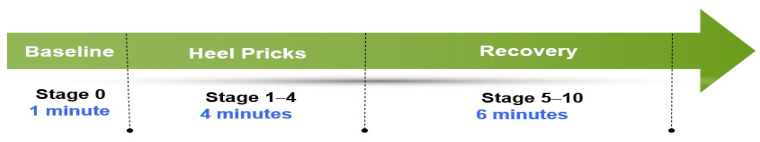
Heel-prick stages (1 min for each stage).

A senior nurse with more than 15 years of clinical experience in neonatal care performed heel pricks. She had been well trained in standard heel-prick procedures for collection of blood samples. She controlled the duration of heel-prick procedure at 3–4 min for all neonates by manipulating the force of gently squeezing their heel during blood collection. The heel prick was initiated at the point in time when the senior nurse touched the heel of the neonate, and the duration ended when her hands left the heel. All heel pricks were divided into eleven 1-min stages: Stage 0 (1 min before heel rick [baseline]), Stages 1–4 (the 1st–4th min of the heel-prick procedure), and Stages 5–10 were recovery (the 1st–6th min after heel-prick ended) (Figure 2).

#### 2.4.2. Intervention

The intervener (the first author) placed the neonate in a quiet, isolated room 30 min before the neonatal screening procedure, avoiding any other disturbances or painful stimuli that might cause excessive fluctuations in biological parameters of pain or behavioral responses. The neonate was placed in a lateral position with towel rolls to support their posture before the heel prick. The neonates in Group 1 (control) received only GT + comforting voice from the intervener, with one hand of the intervener placed on the head of the neonate during and after heel pricks.

Breast milk for the neonates in Groups 2 and 3 was from their own mother, which was manually expressed and collected before breakfast to prevent food intake influencing the odor and then frozen in the nursery. The intervener warmed up the BM before the neonatal screening. Group 2 neonates received the control condition in addition to BMO, provided by placing a cotton ball soaked with 2.5 ml BM 3 cm from the neonates nostril 2 min prior to the heel prick, and was left in place until the collection of the blood sample was completed.

Group 3 neonates received the control condition, BMO as for described for Group 2, and BM taste delivered by syringe feeding. The intervener slowly provided 2.5 mL of BM orally to the neonate from a syringe 1 min before and during the heel-prick procedure.

#### 2.4.3. Study Fidelity

Research fidelity was established by holding bimonthly meetings with the investigators and the senior nurse to confirm the study procedures and discuss any difficulties encountered. The corresponding author checked whether the intervener (the first author) consistently provided BM olfactory and gustatory interventions to the infants in the treatment groups. The first author also consistently collected the data from medical and nursing charts in the manner described by the corresponding author. The corresponding author also discussed with the senior nurse to confirm the consistency of the heel-stick procedures. 

### 2.5. Ethical Considerations

Following approval of the institutional review board of the study site, the third author approached parents of neonates who fulfilled the study criteria and explained the study procedures in detail. Agreement was obtained using a consent form. Case numbers of the demographic questionnaires, voice recordings, and the files of biological and crying data were locked in an area of the primary investigator’s office that was inaccessible to anyone else for keeping the confidentiality of the neonates. Parents of participating neonates were informed that they could quit the study at any time if they felt uncomfortable during the process.

### 2.6. Statistical Analyses

Data were analyzed using the SPSS software version 23.0 (IBM, Armonk, NY, USA). Data for characteristics of the neonates among the three groups were compared using Fisher’s exact test (categorical data) and the nonparametric Kruskal–Wallis test (continuous variables). Data were described using means and standard deviations (SD) for continuous variables and frequencies for categorical data. Generalized estimating equations (GEE) compared effects of the BM olfactory and gustatory interventions on heart rate and SpO_2_ for the different stages of the heel-prick procedure among the three groups using generalized linear models with a two-way interaction (group × stage) by using a first-order autoregressive (AR1) working correlation matrix [39]. The Kaplan–Meier survival analysis and log rank test examined the effects of the BM olfactory and gustatory interventions on the time for stopping crying across the heel-prick procedure, and the Cox proportional hazards model to adjust for the effects of potential confounding variables simultaneously [40]. A *p* value of < 0.05 was considered statistically significant.

## 3. Results

### 3.1. Characteristics of the Neonates

The sample included 114 full-term neonates with a mean GA of 39.08 ± 1.02 weeks and a mean birth weight of 3097.59 ± 360.22 g. Most infants were born by normal spontaneous delivery (66.67%). The mean number of painful experiences was 2.43 ± 0.52, and the mean heel-prick time was 156.62 ± 67.53 s. Baseline clinical characteristics of neonates among the three groups and duration of heel pricks did not differ significantly. Characteristics of the neonates in the three groups are presented in Table 1.

### 3.2. Biological Parameters of Pain

**Figure 3:**
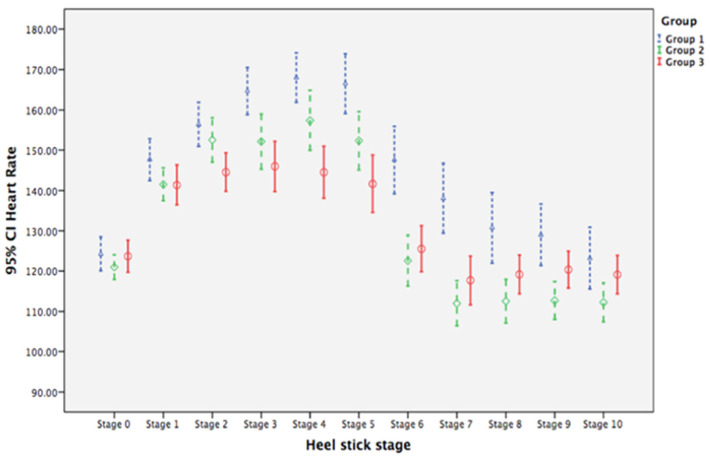
Clustered error-bar graph showing time trends of mean heart rates with 95% confidence intervals (CI) for neonates in the three treatment groups over heel-prick stages. Groups: 1 (blue), gentle touch plus human voice (control condition); 2 (green), control condition + breast milk odor; 3 (red), control condition + breast milk odor + breast milk taste by syringe feeding; Stage 0, baseline (no stimulation); Stages 1 to 4, the 1st to the 4th min during heel prick, respectively; Stages 5 to 10, the 1st to 6th min after completion of heel prick, respectively.

Heart rate at baseline was similar among the three groups (Table 2). For neonates in the control group (Group 1), mean heart rates from stage 1 to stage 7 were, on average, 23.389, 32.126, 40.416, 43.758, 42.258, 23.284, and 13.784 units significantly higher than that at baseline (stage 0), respectively, with all *p* values < 0.05. For neonates in Group 2 (CC + BMO), the changes in mean heart rate at stages 5, 6, 7, 8, and 9 were, on average, 10.905, 21.695, 22.774, 14.932, and 13.037 units significantly lower, respectively, than for neonates in Group 1 at corresponding stages (all *p* values < 0.05). For neonates in Group 3 (CC + BMO + BMTSF), changes in mean heart rate at stages 2, 3, 4, 5, 6, 7, 8 were, on average, 11.274, 18.142, 22.932, 24.300, 21.458, 19.800, and 11.011 units significantly lower, respectively, than for neonates in Group 1 at the corresponding stages (all *p* values < 0.05). The results of GEE analysis suggested that BM sensory interventions reduced the amount of increase in heart rate during a heel-stick procedure compared with GT and nurse voice. Time trends for the changes in mean heart rate are illustrated with clustered error-bar plots (Figure 3). Mean heart rates for neonates in Group 2 and Group 3 were lower than for neonates in the control group (Group 1).

**Figure 4:**
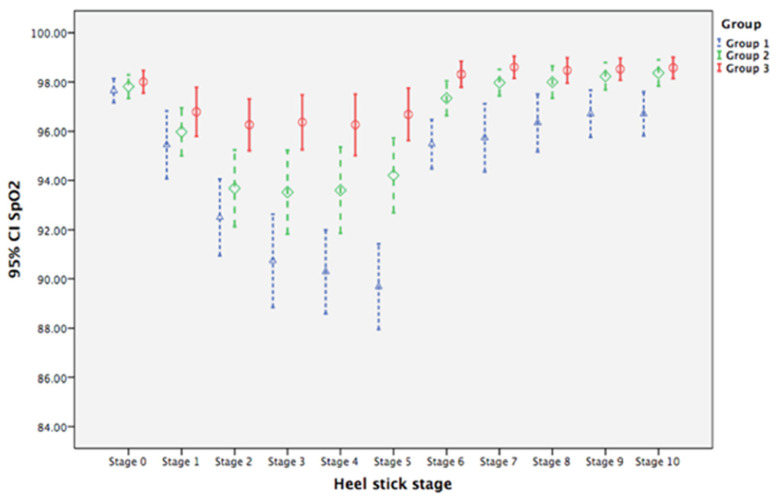
Clustered error-bar graphs showing time trends of mean oxygen saturation (SpO_2_) with 95% confidence intervals (CI) for neonates in the three treatment groups over heel-prick stages. Groups: 1 (blue), gentle touch plus human voice (control condition); 2 (green), control condition + breast milk odor; 3 (red), control condition + breast milk odor + breast milk taste by syringe feeding; Stage 0, baseline (no stimulation); Stages 1 to 4, the 1st to the 4th min during heel prick, respectively; Stages 5 to 10, the 1st to 6th min after completion of heel prick, respectively.

Oxygen saturation levers did not differ at baseline among the three groups (Table 3). The mean levels for Group 1 neonates from Stage 1 to Stage 10 were, on average, 2.205, 5.153, 6.916, 7.363, 7.968, 2.179, 1.916, 1.311, 0.942, and 0.942 units significantly lower, respectively, than that at baseline (Stage 0), with all *p* values < 0.05. For neonates in Group 2, changes in mean SpO_2_ from stage 3 to 10 were, on average, 2.626, 3.153, 4.363, 1.705, 2.074, 1.495, 1.363, and 1.495 units significantly higher, respectively, than for neonates in Group 1 for the corresponding stages (all *p* values < 0.05). For neonates in Group 3, the changes of mean SpO_2_ from Stages 2 to 10 were, on average, 3.405, 5.274, 5.616, 6.642, 2.484, 2.511, 1.774, 1.458, and 1.511 units significantly higher, respectively than for neonates in Group 1 (all *p* values < 0.05; Table 3). GEE analysis suggests that BM olfactory and gustatory interventions prevented neonates from experiencing as much of a decrease in SpO_2_ compared with GT + human voice alone. Figure 4 illustrates the higher mean SpO_2_ level for neonates in Group 2 and Group 3 compared with levels for neonates in Group 1, with mean levels for BM olfactory and gustatory intervention groups returning to baseline levels by Stage 7.

### 3.3. Time to Crying Cessation

**Figure 5:**
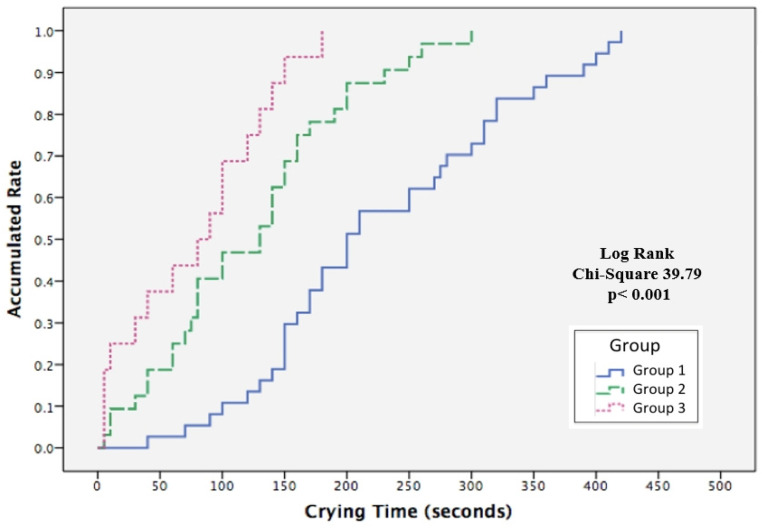
Kaplan–Meier survival analysis comparing the accumulated rate of time to cessation of crying during heel-prick procedures for neonates in the three treatment groups. Groups: 1 (blue), gentle touch plus human voice (control condition); 2 (green), control condition + breast milk odor; 3 (pink), control condition + breast milk odor + breast milk taste by syringe feeding.

Kaplan–Meier log rank test demonstrated significant differences among the three groups time to crying cessation during heel-prick (Figure 5). The median time to stop crying for Groups 1, 2, and 3 were 200, 130e, and 80 s, respectively, with a significant overall cumulative probability (χ^2^ = 39.79, *p <* 0.001). The effect of the BM olfactory and gustatory interventions on the overall cumulative probability curve for crying cessation was assessed by determining the hazard rate ratio (HR) using Cox regression analysis, after adjusting for GA (Table 4). The instantaneous occurrence rates of crying cessation, determined by the HR for Group 2 (CC + BMO) and Group 3 (CC + BMO + BMTSF) were 3.016 (*p* < 0.001) and 6.466 (*p* < 0.001) times, respectively, as compared with Group 1(CC). The BM olfactory and gustatory interventions significantly reduced the time of crying when exposed to procedural pain for neonates in Groups 2 and 3 when compared with Group 1 (CC).

## 4. Discussion

These findings present new knowledge about the effects of olfactory and gustatory sensory stimulation on reducing procedural pain in neonates. Our multisensory interventions of BMO and BMO + BMTSF stabilized heart rate and SpO_2_ and shortened the time to crying cessation across the 11 stages of the heel-prick procedure. Compared with neonates in Group 1 (CC), Group 2 (BMO) and Group 3 (BMO and BMTSF), neonates had smaller mean heart rate changes in Stages 5–9 and Stages 2–9. For neonates in Group 3, this difference was apparent during the heel-prick stages (Figure 3), suggesting that the combination of BMO and BMTSF interventions had a larger calming effect than BM odor alone. Neonates in Groups 2 (Stages 3–10) and Group 3 (Stages 2–10) also had higher mean SpO_2_ levels compared with Group 1 (Figure 4). Changes in heart rate and SpO_2_ are considered biological indicators of pain [8].

These results support not only our hypotheses but also confirm the effects of BMO or BMO + BMTSF on reducing variations in heart rate, SpO_2_, and time to crying cessation. Our study findings echo two meta-analyses on the effects of BMO: one reported that BMO reduced pain [41]; a second reported that BMO affected variations in heart rate, SpO_2_, and crying duration [42]. The results of this study also support reports showing that BM odor has a calming effect, using changes in heart rate and SpO_2_ as biological parameters of pain. One study found that BMO was more effective when compared with formula odor during the heel-prick procedure [43]. Two studies demonstrated a reduction in pain for preterm infants receiving BMO during venipuncture [27,41].

Our findings differ from a study by Küçük Alemdar and Kardaş Özdemir [33] who found no pain scores or crying time differences when BMO and mother’s BM were compared with control groups. Wu et al. [18] reported no significant reduction in stress behaviors or crying duration for preterm infants receiving mother’s BMO with BM taste during venipuncture procedures [18]. A review of studies on nonpharmacological interventions for procedural pain found that BM provided infants with a small amount of analgesia for procedural pain, but it did not completely alleviate pain [34]. These inconsistencies with our results on the effects of BMO and BMTSF on procedural pain may be due to differences in measures of pain assessment, methods of analysis, or maturity of the infants, the pain intensity of the intrusive procedure, and how the BM interventions were provided.

In this study, heart rate and SpO_2_ were continuously monitored and downloaded across the heel-prick procedure. The Kaplan–Meier survival analysis and log rank test analysis of the accumulated rate of crying cessation [40] differ substantially from those of other studies who applied the Mann–Whitney U test [44], Student’s *t* test [45], or one-way ANOVA [46] to compare group differences in crying duration. Our study provides sound evidence for clinicians to provide BMO and BMTSF for neonates who are undergoing heel-prick procedures.

Assessing preterm infants’ responses to painful stimuli is challenging because the immaturity of their nervous system dulls responses to painful stimuli [21]. In our study, all neonates were full term, with full use of their sensory capacities to perceive the odor and taste of BM for self-comfort while encountering pain or stress [21,47]. The study findings demonstrate that the unisensory BM intervention of BMO had a calming effect on neonates by reducing biological and behavioral parameters of pain. The addition of BMTSF to BMO revealed an additive effect for stabilizing neonates’ heart rate, increasing mean levels of SpO_2_, and shortening the time to crying cessation.

Most studies continuously provided the mother’s BMO [27,33,42,43], or breastfeeding [28,44,48] to neonates during painful procedures, which effectively reduced pain and crying time. Our study continuously provided BMO and BMTSF to the neonates before and during the heel-prick procedures by slowly dripping BM into the mouth from a syringe. The findings of this study also echo a report that analgesic effects of BM were similar to sucrose during venipuncture [32]. Two other studies used multisensory interventions. One combined the odor and taste of BM, mother’s heartbeat sound, and NNS to facilitate pain recovery during venipuncture [18]. The other study reported that oral BM needed to be combined with NNS and tucking to generate significant analgesic effects during heel-prick procedures [49].

For breast feeding neonates, the provision of BMO and BMTSF supports the recommendations of the BFHI, advocating for exclusive breastfeeding and avoiding sucrose and NNS in relieving pain and stress [20]. This study also overcame the limitations of the BFHI by using mother’s BM for olfactory and gustatory interventions to stabilize biological parameters of pain and for shortening the time to crying cessation for neonates while undergoing painful procedures. The effects of BMO and BMTSF also expand the benefits of BM for pain relief, which could guide clinicians to modify neonatal care practices; in the past, procedural pain in neonates undergoing intrusive procedures may have been ignored. The evidence in this study can enable better pain management in neonatal clinical practice.

### 4.1. Clinical Implications

Our results add to a growing body of evidence suggesting that BM olfactory and gustatory interventions such as BMO and BMTSF can provide nonpharmacological analgesia in neonates undergoing short painful procedures. The study findings could guide clinicians to provide BM odor on a cotton ball and BM taste via slow syringe feeding for pain relief in neonates during and after heel-prick procedures. The use a slow drip from the syringe was easily implemented for the BMTSF procedure. Clinicians in neonatal and maternal care should encourage mothers to express BM and should allow the neonate to suck the mother’s breast as early as possible after birth to stimulate lactation. In addition, providing mothers with information about analgesic mechanisms and effects of BM on pain relief for neonates and how to facilitate lactation and collect BM should be included in clinical care. A small amount of BM odor (2.5 mL) and BMTSF (2.5 mL) provided to neonates can relieve the biobehavioral response to pain without using pacifiers and sucrose, and it promotes breastfeeding for neonates, infant health, and growth.

### 4.2. Strengths, Limitations, and Recommendations

The strengths of this study include a randomized, controlled trial design; maintaining the intervention integrity and consistency, which included the first author being well-trained in neonatal car and providing multisensory BM interventions; and having two different researchers as the intervener and outcomes measurer to minimize measurement bias. Biological parameters were assessed with a valid oximetry monitor, and the interrater reliability for measuring time to crying cessation was 96%. Furthermore, a senior nurse executed the heel-prick procedure according to the standard protocols, and the heel-prick duration was controlled within 4 min.

In spite of the strengths, the study had some limitations. First, this study measured only heart rate, SpO_2_, and crying during a short-term painful procedure. Future studies could consider other outcomes or painful procedures to strengthen the study findings. Second, the studied population included healthy infants; studies could also explore the effects of BM olfactory and gustatory interventions on preterm infants or on full-term infants with illness. Finally, future research could be conducted to gather additional data and more evidence to encourage clinicians to incorporate multiple sensory interventions (comforting human voice, gentle touch, BMO, and BMTSF) as routine care during heel-prick procedures.

## 5. Conclusions

Our results confirm the research hypothesis that neonates receiving either BMO or BMO plus BMTSF would have reductions in changes of heart rates and SpO_2_ and shorter time to crying cessation during and after a heel-prick procedure compared with neonates receiving GT and human voice interventions. The study findings provide guidance for clinicians offering humanistic and atraumatic care to improve health outcomes in neonates. Clinicians could implement BMO or BMO plus BMTSF to stabilize the infant’s biobehavioral responses to short-term painful procedures. Breast milk can not only provide nutrients but can also buffer pain and biobehavioral stress while undergoing intrusive procedures. Clinicians do not need to use pacifiers or sucrose to relieve pain. However, replication is necessary to strengthen the findings before recommending the intervention’s incorporation into routine care for other invasive procedures.

## Figures and Tables

**Table 1:** Baseline characteristics and duration of heel-prick procedure for neonates in the three groups (N = 114).

Variable	Group 1 (CC)				Group 2 (CC+BMO)				Group 3 (CC+BMO+BMTSF)				*p*
	(*n* = 38)				*n* = 38				*n* = 38				
	Mean	SD	*n*	%	Mean	SD	*n*	%	Mean	SD	*n*	%	
**Baseline Measures**													
Gestational age (weeks)	39.36	0.99			38.98	1.04			38.89	0.99			0.066 ^1^
Birth weight (g)	3120.92	355.69			3105.39	385.47			3066.45	345.80			0.836 ^1^
Delivery type													0.128 ^2^
Normal spontaneous			30	78.95			22	57.89			24	63.16	
Caesarean			8	21.05			16	42.11			14	36.84	
Sex													0.656 ^2^
Male			17	44.74			21	55.26			19	50.00	
Female			21	55.26			17	44.74			19	50.00	
Apgar score: 1 min	7.82	0.39			7.84	0.37			7.87	0.34			0.822 ^1^
Apgar score: 5 min	9.00	0.00			8.97	0.16			8.97	0.16			0.604 ^1^
Times of painful experiences	2.53	0.56			2.39	0.50			2.37	0.49			0.419 ^1^
Oxygen saturation (%)	97.65	1.50			97.82	1.46			98.01	1.38			0.543 ^1^
Heart rate (bpm)	124.27	12.79			120.99	9.34			123.70	12.14			0.596 ^1^
Heel-prick duration (s)	163.68	71.08			160.53	68.60			145.66	63.08			0.468 ^1^

SD, standard deviation; CC, control condition: gentle touch plus human voice; BMO, breast milk odor; BMTSF, breast milk taste syringe fed. ^1^ Kruskal–Wallis test. ^2^ Fisher’s exact test.

**Table 2:** Comparison of changes in heart rates among neonates in the three treatment groups across the 11 stages of the heel-prick procedure: Analysis using generalized estimating equation with multiple linear regression.

Variable	*B*	*SE*	Wald χ^2^	*p*	95% CI	
					Lower	Upper
**Group Effects**						
Group 3 vs. Group 1	−0.568	2.823	0.041	0.840	−6.102	4.965
Group 2 vs. Group 1	−3.279	2.535	1.673	0.196	−8.248	1.690
**Stage Effects**						
Stage 10 vs. Stage 0	−1.084	3.445	0.099	0.753	−7.837	5.669
Stage 9 vs. Stage 0	4.732	3.854	1.507	0.220	−2.822	12.285
Stage 8 vs. Stage 0	6.468	4.350	2.211	0.137	−2.058	14.995
Stage 7 vs. Stage 0	13.784	4.531	9.256	0.002	4.904	22.664
Stage 6 vs. Stage 0	23.284	4.325	28.987	<0.001	14.808	31.761
Stage 5 vs. Stage 0	42.258	3.849	120.557	<0.001	34.715	49.801
Stage 4 vs. Stage 0	43.758	3.157	192.061	<0.001	37.569	49.946
Stage 3 vs. Stage 0	40.416	3.292	150.740	<0.001	33.964	46.868
Stage 2 vs. Stage 0	32.126	3.028	112.592	<0.001	26.192	38.060
Stage 1 vs. Stage 0	23.389	2.640	78.510	<0.001	18.216	28.563
**Interaction Effects**						
**Group 2 × Stage**						
Group 2 × Stage 10	−7.668	3.978	3.717	0.054	−15.464	0.127
Group 2 × Stage 9	−13.037	4.2518	9.405	0.002	−21.369	−4.705
Group 2 × Stage 8	−14.932	4.836	9.534	0.002	−24.409	−5.454
Group 2 × Stage 7	−22.774	5.119	19.792	<0.001	−32.807	−12.740
Group 2 × Stage 6	−21.695	5.108	18.041	<0.001	−31.706	−11.684
Group 2 × Stage 5	−10.905	5.204	4.391	0.036	−21.106	−0.705
Group 2 × Stage 4	−7.379	4.837	2.327	0.127	−16.859	2.101
Group 2 × Stage 3	−9.274	4.7261	3.850	0.050	−18.537	−0.011
Group 2 × Stage 2	−0.589	4.062	0.021	0.885	−8.552	7.373
Group 2 × Stage 1	−2.853	3.370	0.716	0.397	−9.458	3.753
**Group 3 × Stage**						
Group 3 × Stage 10	−3.511	3.876	0.820	0.365	−11.108	4.087
Group 3 × Stage 9	−8.063	4.128	3.816	0.051	−16.153	0.027
Group 3 × Stage 8	−11.011	4.634	5.646	0.017	−20.092	−1.929
Group 3 × Stage 7	−19.800	4.909	16.268	<0.001	−29.422	−10.178
Group 3 × Stage 6	−21.458	4.716	20.706	<0.001	−30.700	−12.216
Group 3 × Stage 5	−24.300	4.843	25.178	<0.001	−33.792	−14.808
Group 3 × Stage 4	−22.932	4.101	31.270	<0.001	−30.969	−14.894
Group 3 × Stage 3	−18.142	4.146	19.149	<0.001	−26.268	−10.016
Group 3 × Stage 2	−11.274	3.783	8.882	0.003	−18.688	−3.860
Group 3 × Stage 1	−5.721	3.544	2.606	0.106	−12.667	1.225

SE, standard error; CI, confidence interval; Group 1, control condition (CC) = gentle touch (GT) and human voice; Group 2, CC + breast milk odor (BMO); Group 3, CC + BMO + BM taste syringe feeding (BMTSF); Stage 0, baseline (no stimulation); Stage 1–4, the 1st to 4th min during heel prick, respectively; Stages 5 to 10, the 1st to 6th min after completion of heel prick, respectively.

**Table 3:** Comparisons of changes in oxygen saturation (SpO_2_) for neonates in the three treatment groups across the 11 stages of the heel-prick procedure: analysis using generalized estimating equation with multiple linear regression.

Variable	*B*	*SE*	Wald χ^2^	*p*	95% CI	
					Lower	Upper
**Group Effects**						
Group 3 vs. Group 1	0.358	0.327	1.201	0.273	−0.282	0.998
Group 2 vs. Group 1	0.163	0.335	0.237	0.626	−0.494	0.820
**Stage Effects**						
Stage 10 vs. Stage 0	−0.942	0.437	4.638	0.031	−1.799	−0.085
Stage 9 vs. Stage 0	−0.942	0.474	3.957	0.047	−1.870	−0.014
Stage 8 vs. Stage 0	−1.311	0.580	5.112	0.024	−2.447	−0.174
Stage 7 vs. Stage 0	−1.916	0.688	7.749	0.005	−3.265	−0.567
Stage 6 vs. Stage 0	−2.179	0.463	22.186	<0.001	−3.086	−1.272
Stage 5 vs. Stage 0	−7.968	0.870	83.917	<0.001	−9.673	−6.264
Stage 4 vs. Stage 0	−7.363	0.797	85.260	<0.001	−8.926	−5.800
Stage 3 vs. Stage 0	−6.916	0.896	59.519	<0.001	−8.673	−5.159
Stage 2 vs. Stage 0	−5.153	0.738	48.778	<0.001	−6.599	−3.707
Stage 1 vs. Stage 0	−2.205	0.538	16.799	<0.001	−3.260	−1.151
**Interaction Effects**						
**Group 2 × Stage**						
Group 2 × Stage 10	1.495	0.504	8.810	0.003	0.508	2.482
Group 2 × Stage 9	1.363	0.517	6.965	0.008	0.351	2.376
Group 2 × Stage 8	1.495	0.637	5.508	0.019	0.246	2.743
Group 2 × Stage 7	2.074	0.734	7.982	0.005	0.635	3.512
Group 2 × Stage 6	1.705	0.541	9.956	0.002	0.646	2.765
Group 2 × Stage 5	4.363	1.0856	16.154	<0.001	2.235	6.491
Group 2 × Stage 4	3.153	1.0889	8.382	0.004	1.018	5.287
Group 2 × Stage 3	2.626	1.1474	5.239	0.022	0.377	4.875
Group 2 × Stage 2	1.021	1.0021	1.038	0.308	−0.943	2.985
Group 2 × Stage 1	0.363	0.6773	0.288	0.592	−0.964	1.691
**Group 3 × Stage**						
Group 3 × Stage 10	1.511	0.470	10.323	0.001	0.589	2.432
Group 3 × Stage 9	1.458	0.501	8.457	0.004	0.475	2.440
Group 3 × Stage 8	1.774	0.601	8.714	0.003	0.596	2.951
Group 3 × Stage 7	2.511	0.715	12.321	<0.001	1.109	3.912
Group 3 × Stage 6	2.484	0.498	24.850	<0.001	1.507	3.461
Group 3 × Stage 5	6.642	0.986	45.398	<0.001	4.710	8.574
Group 3 × Stage 4	5.616	0.948	35.124	<0.001	3.759	7.473
Group 3 × Stage 3	5.274	0.996	28.056	<0.001	3.322	7.225
Group 3 × Stage 2	3.405	0.868	15.409	<0.001	1.705	5.105
Group 3 × Stage 1	0.984	0.685	2.064	0.151	−0.359	2.327

*SE*, standard error; CI, confidence interval; Group 1, control condition (CC) = gentle touch (GT) and human voice; Group 2, CC + breast milk odor (BMO); Group 3, CC + BMO + BM taste syringe feeding (BMTSF); Stage 0, baseline (no stimulation); Stage 1–4, the 1st to 4th min during heel prick, respectively; Stages 5 to 10, the 1st to 6th min after completion of heel prick, respectively.

**Table 4:** Comparison of group effects on crying cessation during heel prick for neonates using Cox regression after adjusting for gestational age.

Variable	*B*	*SE*	Wald χ^2^	*p*	HR	95% CI	
						Lower	Upper
**Group Effects**							
Group 2 vs. Group 1	1.104	0.276	15.982	<0.001	3.016	1.755	5.182
Group 3 vs. Group 1	1.866	0.355	27.689	<0.001	6.466	3.226	12.958
**Infant Characteristics**							
Gestational age	−0.323	0.125	6.692	0.010	0.724	0.567	0.925

*SE*, standard error; HR, hazard rate ratio; CI, confidence interval; Group 1, control condition (CC) = gentle touch (GT) and human voice; Group 2, CC + breast milk odor (BMO); Group 3, CC + BMO + BM taste syringe feeding (BMTSF).

## Data Availability

Data are available from the corresponding author on request.
